# Bacterial biofilm functionalization through Bap amyloid engineering

**DOI:** 10.1038/s41522-022-00324-w

**Published:** 2022-08-01

**Authors:** Leticia Matilla-Cuenca, Agustina Taglialegna, Carmen Gil, Alejandro Toledo-Arana, Iñigo Lasa, Jaione Valle

**Affiliations:** 1 Instituto de Agrobiotecnología (IDAB). CSIC- Gobierno de Navarra, Mutilva, Spain; 2 Laboratory of Microbial Pathogenesis, Navarrabiomed, Hospital Universitario de Navarra (HUN), Universidad Pública de Navarra (UPNA), IdiSNA, Pamplona, Spain; 3 Present Address: The Campus 4 Crinan Street London N1, London, UK

**Keywords:** Biofilms, Applied microbiology

## Abstract

Biofilm engineering has emerged as a controllable way to fabricate living structures with programmable functionalities. The amyloidogenic proteins comprising the biofilms can be engineered to create self-assembling extracellular functionalized surfaces. In this regard, facultative amyloids, which play a dual role in biofilm formation by acting as adhesins in their native conformation and as matrix scaffolds when they polymerize into amyloid-like fibrillar structures, are interesting candidates. Here, we report the use of the facultative amyloid-like Bap protein of *Staphylococcus aureus* as a tool to decorate the extracellular biofilm matrix or the bacterial cell surface with a battery of functional domains or proteins. We demonstrate that the localization of the functional tags can be change by simply modulating the pH of the medium. Using Bap features, we build a tool for trapping and covalent immobilizing molecules at bacterial cell surface or at the biofilm matrix based on the SpyTag/SpyCatcher system. Finally, we show that the cell wall of several Gram-positive bacteria could be functionalized through the external addition of the recombinant engineered Bap-amyloid domain. Overall, this work shows a simple and modulable system for biofilm functionalization based on the facultative protein Bap.

## Introduction

Many amyloids found in nature, including those produced by some bacterial species, carry out relevant biological purposes and therefore they are named as “functional amyloids”^1^. Functional amyloids are important components of the biofilm matrix in many bacteria. In the amyloid conformation, the proteins become more resistant to proteases and harsh denaturing conditions, conferring stability to the matrix and protection to the embedded bacteria^2–5^. Furthermore, polymerization into amyloid-like fibers occurs in the absence of energy supply. An important feature of amyloids in biofilms is their propensity to interact with other matrix components and modify the viscoelastic properties of the extracellular matrices, which could aid to resist environmental fluctuations^6,7^. Considering the intrinsic properties of the amyloids, researches have made use of these structures in order to construct tunable living materials or functionalized biofilms for many technical and applicative biotechnological purposes^8–12^. A well characterized functional amyloid system from bacterial biofilm matrix is the curli fimbriae from *Escherichia coli* and other enterobacteria^13,14^. Curli amyloids are formed by the self-assembly of the major subunit CsgA, where the minor subunit CsgB accelerates CsgA assembly. The curli subunit CsgA has been genetically engineered with different proteins, peptides tags and bioactive domains to obtain functionalized biomaterials and biofilms for different purposes such as nanowires and nanorods, patternable materials, biomimetic mineralization, medical hydrogels and modulators of host-microbes interactions^15–20^. However, the curli system has not only size limitations for the peptides that can be fused to CsgA but also conformational restrictions^19^. This means that the structural properties of the fusion tags can block their secretion through the pore constituted by CsgG^19^. Interestingly, size and conformational limitations can be circumvented using covalent immobilization of proteins through the SpyTagSpyCatcher system in which a 13-amino-acid peptide (SpyTag) forms a highly specific covalent bond with a 15-kDa protein (SpyCatcher)^11,21^. The first component (SpyTag) can be fused to CsgA and expressed through the curli machinery and the second component (SpyCatcher) can be fused to the functional tags of interest^11,12,20,22^.

Another group of functional amyloids involved in building the biofilm matrix is represented by the biofilm associated protein (Bap) of *S. aureus*^23^. Bap belongs to a large family of proteins expressed by Gram-positive and Gram-negative bacteria^24^. Among staphylococci, Bap is expressed by *S. aureus* and coagulase-negative staphylococci^25^. Bap is a multi-domain protein including the N-terminal secretory signal peptide in the region A, two EF-hand calcium binding motifs and several amyloidogenic peptides in the region B^26,27^. Subsequently, the core domain of the protein (region C) that is formed by 14 mostly identical repeats and the C-terminus carrying the LPXTG motif. Bap is covalently linked to the cell wall by a mechanism requiring both the N-terminal secretory signal peptide and the LPXTG motif sorting signal. Once the protein is exposed on the cell surface it promotes the adhesion of *S. aureus* to biotic and abiotic surfaces. When bacteria encounter acidic environments, Bap is processed liberating a N-terminal fragment that includes the region A and B, which adopts a β-sheet-rich structure and establishes amyloid-like fibers that mediate bacterial interactions and modulate biofilm strength. On the contrary, when bacteria are exposed to neutral pH, Bap is displayed at the cell surface and does not mediate biofilm formation^26,28^. This pH-regulated dual behavior has been also shown for the enterococcal surface protein (Esp) of *Enterococcus faecalis*, which is a Bap ortholog^29^. In addition, amyloid peptides derived from the Bap sequence of *S. epidermidis* have been described^30^. It has been proposed that they mediate protein–protein interactions and the tertiary structure of Bap in *S. epidermidis*. Interestingly, amyloid sequences are also present in Bap-orthologs from many bacterial species suggesting that the mechanism of amyloid-like aggregation to build the biofilm matrix might be widespread among this family of proteins^29^.

Some amyloidogenic proteins, including Bap, play dual roles as bacterial adhesins and scaffold components of the biofilm matrix. Therefore, they are referred as facultative amyloids. These proteins may have multiple domains, some of them involved in biofilm formation^31^, which can be engineered to construct functionalized biofilms for biotechnological purposes^32^. In this work we take advantage of Bap properties to confer programmable functions in the globular and amyloid conformation of the protein. We created a battery of functional domains, including metal ions binding (6xHis, Metallothionein 1-MT1), fluorescent (SNAP-tag and mCherry), adhesive (Mefp3) and conjugative (SpyTag/SpyCatcher) domains fused to Bap and showed that their presence did not affect Bap capacity to mediate adhesin function or to multimerize into amyloid-like fibers. By using fluorescent tags, we validated the pH-mediated dynamics of Bap amyloid fiber formation. Moreover, we built a tool modulated by the pH for covalent immobilization of molecules at the cell surface of the bacteria or at the biofilm matrix using the SpyTag/SpyCatcher system. Our results also showed the functionalization of unmodified Gram-positive bacteria through the exogenous addition of functionalized amyloid domains, opening the possibility to use these functional proteins beyond current applications.

## Results

### Engineering of Bap with fusion domains does not affect protein functionality

**Fig. 1:**
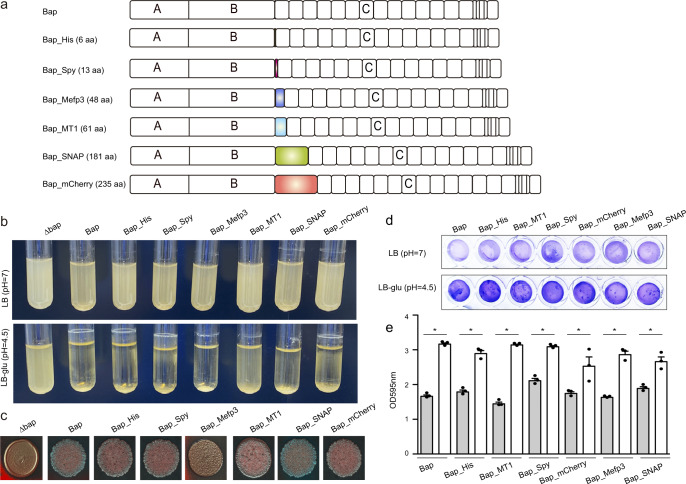
Construction of engineered Bap with fusion domains. **a** Collection of tags fused to the end of the amyloid domain B of Bap: His-tag (6 aa); SpyTag (13 aa); Mefp3 (48 aa); MT1 (61 aa); SNAP-tag (181 aa); mCherry (235 aa). **b** *S. aureus* expressing engineered Bap grow in suspension when they are cultured in LB media (upper panel) or form bacterial clumps in LB with glucose 0.5% (w/v) (LB-glu) overnight cultures grown under shaken conditions (200 rpm) at 37 °C (bottom panel). **c** Colony morphologies of *S. aureus* expressing engineered Bap on Congo red agar after 24 h of incubation. **d** Biofilms formed by *S. aureus* expressing engineered Bap were stained with crystal violet. For biofilm formation, bacteria were cultured in LB or LB-glu overnight at 37 °C in microtiter plates under static conditions. **e** Biofilms formed in LB (grey bars) or LB-glu (white bars) were quantified by solubilizing the crystal violet with alcohol-acetone and determining the absorbance at 595 nm. The error bars represent standard deviations of 3 repetitions. Statistical significance differences were determined using non-parametric one-tail Mann Whitney test: *P < 0.05.

We evaluated the functionalization potential of self-polymerizing Bap based on its ability to tolerate fusion to different protein domains (Fig. 1a). For that, we constructed a collection of *S. aureus* strains expressing Bap functionalized with different tags. Selected tags ranged from 6 to 235 amino acids in length and encoded for a wide variety of functions such as binding to various metal ions (6xHis, Metallothionein 1-MT1), interaction to specific partners (SpyTag, SNAP), adhesive proteins (Mefp3), fluorescence (mCherry). Each tag was chromosomally fused to the end of the amyloid domain B of Bap in *S. aureus* V329 strain, without leaving behind an antibiotic resistance marker (Fig. 1a).

Next, we evaluated whether the addition of these tags to the protein affected Bap-mediated multicellular behavior. For that, *S. aureus* was grown in LB media containing glucose. Entry into the stationary phase is accompanied by a decrease in pH due to the accumulation of acidic by-products from glucose fermentation. Under these culture conditions, *S. aureus* expressing Bap derivative proteins retained the capacity to form bacterial clumps and formed a biofilm on microtiter plates (Fig. 1b, d). Similar phenotypes were observed for *S. aureus* V329 expressing the intact Bap protein. In contrast, the Δ*bap* mutant, which was used as a negative control, was unable to form biofilm. When Bap derivative strains were grown in LB media, where the pH is maintained as neutral, bacteria failed to form cell clumping and biofilm development as expected (Fig. 1b, d). These results showed that Bap biofilm formation capacity tuned by pH was not affected by tag addition. Finally, we evaluate the colony morphology on CR plates, as an indicative of amyloid production^33^. *S. aureus* expressing Bap derivative proteins formed rough colonies on CR agar (Fig. 1c), indicating that amyloid formation is maintained. Only cells expressing Bap-Mefp3 showed a smoother colony morphology compared with cells expressing the intact protein (Fig. 1c). Taken together all these results showed that Bap can be functionalized with several tags of different sizes without affecting Bap-mediated multicellular behavior.

### Use of fluorescent tags to visualize in vivo formation of Bap amyloid-dependent matrices

**Fig. 2:**
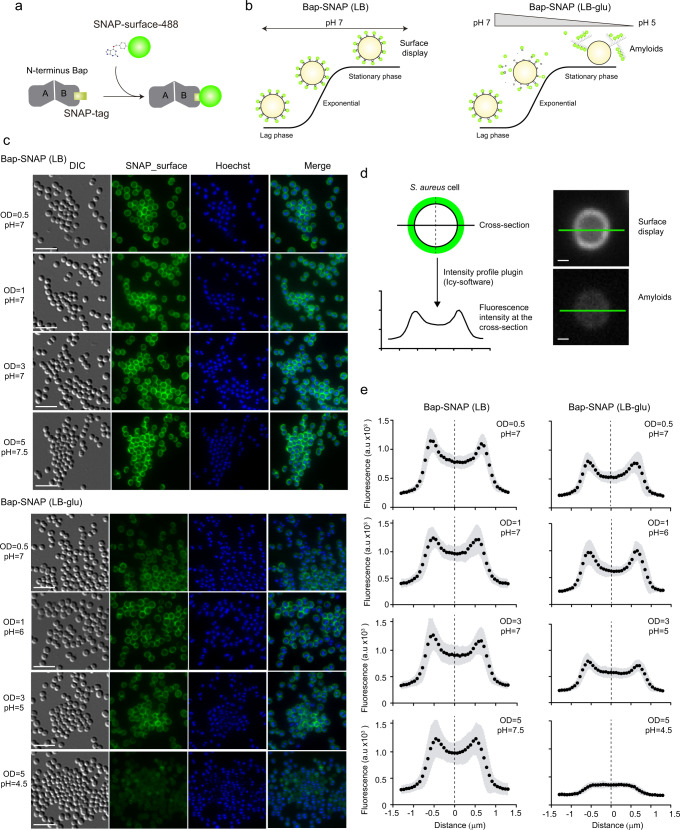
SNAP-tag is an efficient tool for visualize the effect of pH on Bap-mediated multicellular behavior. **a** Schematic representation of Bap-SNAP labelling mechanism. **b** Scheme illustrating the effect of pH on Bap-mediated multicellular behavior. When *S. aureus* expressing Bap-SNAP is cultured in LB the pH of the media keeps neutral along the growth curve. In this culture conditions Bap is expressed at the cell surface of the bacteria. When *S. aureus* expressing Bap-SNAP is cultured in LB with glucose 0.5% (w/v) (LB-glu) the pH of the media is acidified when bacteria enter in stationary phase. Bap N-terminus is processed and N-terminal fragments start to self-assemble and ultimately form amyloid fibers that mediate cell-to-cell contacts. **c** Immunofluorescence showing localization of Bap in *S. aureus* expressing Bap-SNAP grown in LB and LB-glu media at 37 °C, 200 rpm. Samples were taken at different points of the growth curve. Cells were labelled with SNAP-surface 488 substrate and Hoechst. The fluorescence of SNAP-surface 488 and Hoechst, the combination of both signals (merge panels) and the differential interference contrast (DIC) images are shown. Scale bar of panels represents 5 μm. **d** Schematic representation of the fluorescence intensity profile of cross-section of individual cells. The fluorescence intensity was determined using the Intensity profile plugin of Icy-software. This plugin calculates the intensity value of each pixel along a cross-section line of 3 μm that was drawn on individual cells. *S. aureus* Bap-SNAP individual cell grown in LB (upper right panel) and in LB-glu (lower right panel) with cross-section draw was shown. Scale bar of panels represents 0.5 μm. **e** Graphs correspond to the mean of the intensity profiles of cross-sections cells (*n* = 40). Gray shadow corresponds to standard deviation of the mean.

**Fig. 3:**
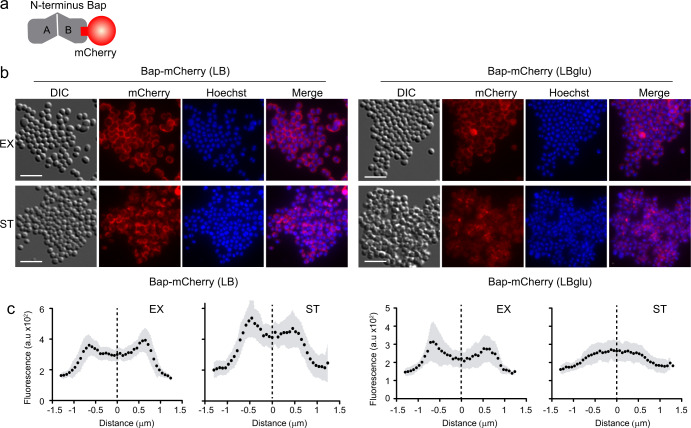
Direct fluorescent visualization of Bap-mediated multicellular behavior. **a** Schematic representation of Bap-mCherry fusion. **b** Immunofluorescence showing localization of Bap in *S. aureus* expressing Bap-mCherry grown in LB and LB-glu media at 37 °C, 200 rpm until exponential phase (EX) (OD = 0.5) and stationary phase (ST) (OD = 5). The fluorescence of mCherry and Hoechst, the combination of both signals (merge panels) and the differential interference contrast (DIC) images are shown. Scale bar of panels represents 5 μm. **c** Graphs correspond to the mean of the intensity profiles of cross-sections cells (*n*~20). Gray shadow corresponds to standard deviation of the mean.

Bap is displayed at the cell surface from where it plays dual roles as bacterial adhesin and as a scaffold component of the biofilm matrix. In an effort to visualize and discriminate between the amyloid-forming and non-amyloid forming Bap-related phenotypes, we used the SNAP-tag labeling approach. SNAP-tag covalently reacts with O6-benzylguanine (BG) substrate with an adapted synthetic label fluorophore (Fig. 2a). Under acidic conditions, Bap is processed releasing the N-terminal domain, which acquires a β-sheet-rich structure that mediates amyloid-like fibers as biofilm matrix scaffolds, while under neutral pH Bap is maintained attached to the cell surface (Fig. 2b). Therefore, to monitor the expression and localization of Bap including the SNAP tag on the *S. aureus* surface, bacteria grown in LB and LB-glu along the growth curve were labelled with the SNAP-surface Alexa Fluor 488 cell-impermeable substrate. As negative control we included the *S. aureus* V329 strain expressing wild-type Bap (Supplementary Fig. 1). As expected, Fig. 2c shows that labeling was successfully accomplished. On the one hand, bacterial cells grown in LB displayed the characteristic well-defined surface localization of Bap in all the points of the growth curve. On the other hand, bacteria grown in a medium with glucose showed two different labeling patterns depending on the growth phase. A well-defined surface labeling of Bap was observed in bacteria at exponential and late exponential phase (OD_600nm_ = 0.5 and 1) where the pH of the media is still neutral (pH = 7 and 6 respectively). In contrast, it displayed a more dispersed labeling with green background when bacteria entered in stationary phase (OD_600nm_ = 3 and 5; pH = 5 and 4.5 respectively), which corresponded to amyloid formation phase. As expected, no fluorescence was observed in *S. aureus* V329 cells grown in LB or LB-glu labelled with SNAP-surface Alexa Fluor 488 (Supplementary Fig. 1). The intensity profile tool in Icy was used to generate fluorescence intensity as a function of the distance of cross-section cells. The fluorescence intensity of *S. aureus* cells grown in LB showed an increase fluorescence signal located 0.5–0.8 μm on each side of the middle of the cross-section line (Fig. 2d, e). These results supported the microscopy analysis and confirmed that Bap was localized surrounding the cells in all the analyzed points of the growth curve. On the other hand, Bap localization changed in *S. aureus* Bap-SNAP cells grown in LB-glu. When the pH was acidified Bap was delocalized from the cell surface and it displayed a reduce intensity projection (Fig. 2d, e). Taking together these results confirmed that functionalized Bap is displayed tightly bound at the cell surface or dispersed in the biofilm matrix depending on the pH. To corroborate the previous result without the need of an exogenous substrate and to demonstrate the dual role of Bap, we used Bap including the fluorescent mCherry tag for its direct visualization on cell surface (Fig. 3a). As shown in Fig. 3b, fluorescence emitted by the mCherry protein can be observed all around the bacteria surface in exponential phase (OD_600nm_ = 0.5) both in the presence or absence of glucose. When bacteria enter the stationary phase (OD_600nm_ = 5), the fluorescent pattern remains mainly localized around bacteria at neutral pH. When the pH becomes acidic, mCherry fluorescence is scattered in the matrix and Bap forms amyloid fibers. Quantification of the fluorescence intensity profile of cross-section from single-cells confirms the delocalization of Bap from cell surfaces when the pH becomes acidic (Fig. 3c). All together, these results validate that Bap localization on the cell surface changes in response to the pH of the medium through the transition from a globular to an amyloid conformation.

### Real-time modulation of Bap-amyloid formation through acidification of pH

**Fig. 4:**
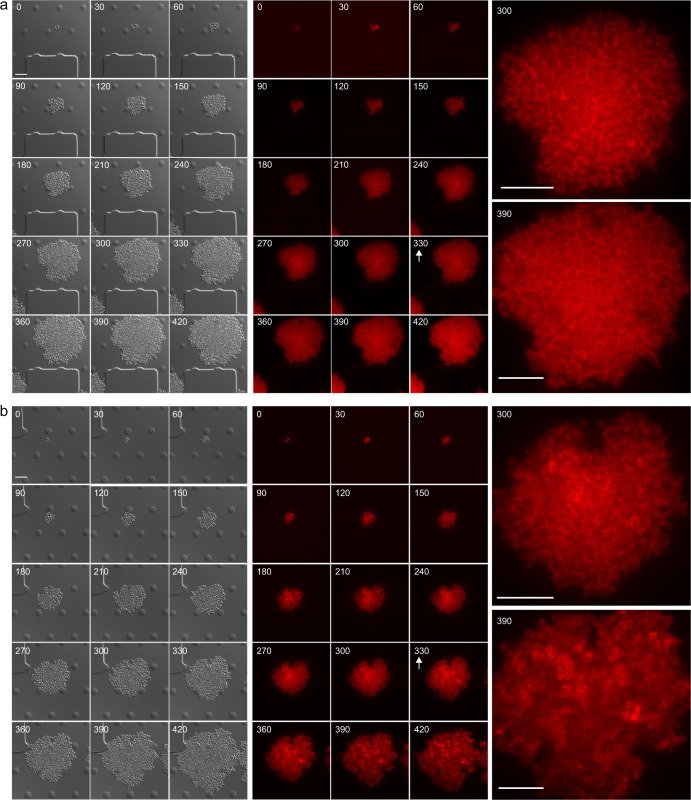
Real-time modulation of Bap-amyloid assembly through acidifying the growth medium. Time-lapse fluorescence microscopy was performed to monitor mCherry expression at single-cell level in *S. aureus* Bap-mCherry. In order to visually compare image patterns, the histogram range of each image was modified accordingly. Bacteria were grown at 37 °C in CellAsic microfluidic plates with a continuous flow of LB for 330 min. Next, bacteria were challenged with LB (neutral pH) **a** or with acidified LB **b** for 90 min. Arrows show the point in which the media was changed. Images were taken in 30 min intervals. The mCherry fluorescence and the differential interference contrast (DIC) images are shown. Right panels show the magnification of images taken 30 min before and 60 min after media challenge. Scale bar of panels represents 5 μm.

Considering that Bap amyloids are modulable through pH changes, we decided to investigate amyloid fiber formation in a dynamic way and in real time, by monitoring Bap-mCherry expression at single-cell level using time-lapse fluorescence microscopy. *S. aureus* Bap-mCherry strain was grown in CellAsic microfluidic plates at 37 °C with a continuous flow of LB medium for 330 min, allowing the formation of microcolonies. Then, to induce amyloid formation, microcolonies were challenged with acidified LB, which was previously obtained from cultures grown in LB-glu until stationary phase, or LB as negative control. Images were recorded every 30 min in separate fields to avoid photobleaching and mCherry fluorescence was monitored in the course of the 7-h experiment. Figure 4a shows that, when microcolonies were grown in LB, the fluorescence pattern of mCherry remains located all the time around the cells, which formed a sort of “cellular honeycomb” due to the 2D arrangement of the microcolony forced by the microfluidic channel. In contrast, when the media was changed to acidified LB (Fig. 4b), the “cellular honeycomb” disassembled and the fluorescence was diffused throughout the bacteria extracellular space, suggesting that Bap is forming amyloid-like structures (see images from 360 to 420 min, Fig. 4b). These results allow us to conclude that Bap expression is homogeneous in each bacterial clone and that amyloid polymerization of Bap can be activated by acidifying the pH of the media.

### Real-time immobilization of SpyCatcher using Bap-Spy

We decided to design a versatile tool for functionalizing Bap-mediated biofilms based on the SpyTag/SpyCatcher system, which offers a highly versatile mechanism to covalently link on any enzyme or recognition molecule to a protein of interest^21^. The SpyTag and the SpyCatcher are peptides of 13 and 138 amino acids respectively, which were engineered by splitting the collagen adhesin domain (CnaB2) of the fibronectin binding protein FbaB of *Streptococcus pyogenes*. The SpyTag forms a robust and highly stable covalent bond with its protein partner, the SpyCatcher, when it is externally added. This linkage is efficient at different pH and temperature ranges and at different buffer solutions^21^.

**Fig. 5:**
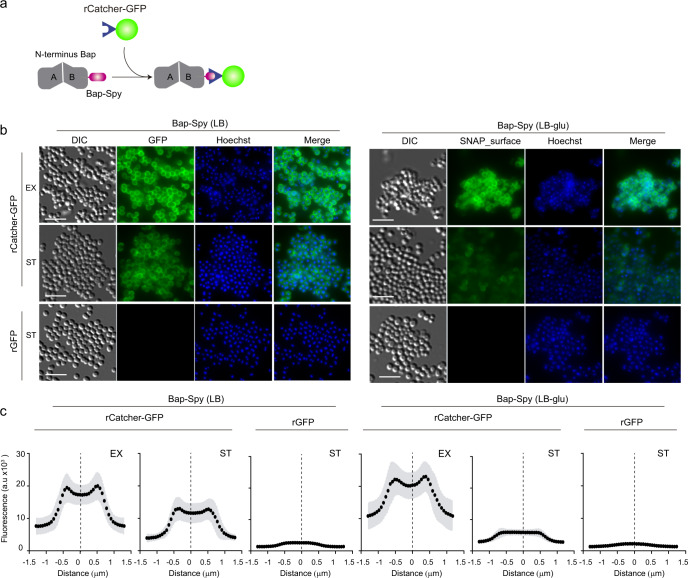
SpyTag/SpyCatcher system can be used to generate a versatile tool for functionalized surface-displayed bacteria or Bap amyloid-like fibers. **a** Schematic representation of Bap-Spy/rCatcher-GFP labelling mechanism. When the protein partner SpyCatcher fused to GFP is added, a covalent bond is formed between Bap-Spy and rCatcher-GFP resulting in fluorescent labeling of Bap. **b** Fluorescence images showing localization of Bap in *S. aureus* Bap-Spy grown in LB and LB-glu media at 37 °C, 200 rpm until exponential phase (EX) (OD = 0.5) and stationary phase (ST) (OD = 5). Cells were probed with purified recombinant rCatcher-GFP and purified rGFP alone (used as a control) followed by nucleic-acid stain Hoechst. The fluorescence of GFP and Hoechst, the combination of both signals (merge panels) and the differential interference contrast (DIC) images are shown. Scale bar of panels represents 5 μm. **c** Graphs correspond to the mean of the intensity profiles of cross-sections cells (*n*~30). Gray shadow corresponds to standard deviation of the mean.

As a proof of concept, the *S. aureus* strain expressing the Bap-Spy protein was grown in LB or LB-glu until exponential or stationary phase. Then, in order to properly detect the covalent linkage between the SpyTag and the SpyCatcher, the cells were incubated with 0.5 mg/ml of purified recombinant SpyCatcher fused to the reporter fluorescent protein GFP (rCatcher-GFP) (Fig. 5a). Binding of rCatcher-GFP to Bap-Spy protein was monitored by fluorescence microscopy (Fig. 5b) and accumulation of the rCatcher-GFP around cells was quantified by Icy software using the Intensity Profile plugin (Fig. 5c). As a negative control, *S. aureus* Bap-Spy cells were incubated with purified untagged rGFP. As illustrated in Fig. 5b, cells grown at neutral pH showed a clear distribution of GFP on the bacterial surface since no amyloid-like aggregates were formed. In contrast, rCatcher-GFP binding developed a more dispersed distribution of the Bap-Spy protein when bacteria grown at acidic pH, indicative of Bap amyloid aggregates acting as biofilm scaffolds. Note that no fluorescent labelling was observed when rGFP (negative control) were added to the cells, confirming the specificity of the labelling (Fig. 5b, c).

**Fig. 6:**
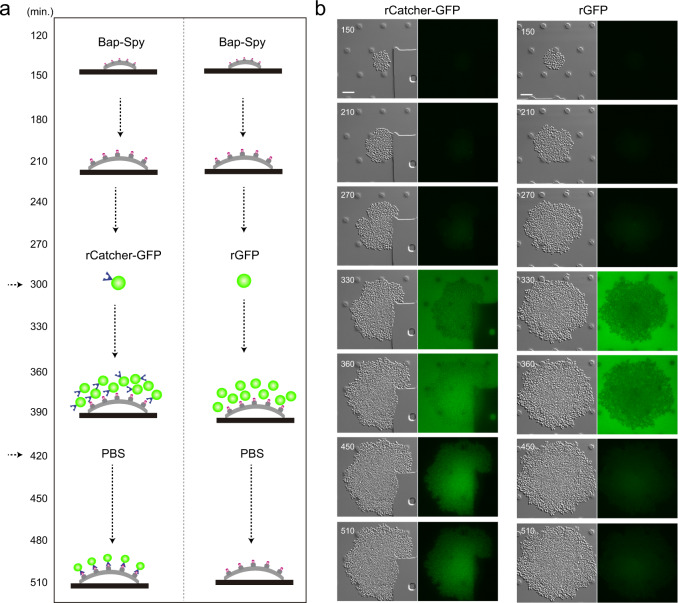
Real time immobilization of epitopes under flow culture conditions using SpyTag/SpyCatcher system. **a** Schematic diagram of the experiment. *S. aureus* Bap-Spy was grown in CellAsic microfluidic plates at 37 °C with a continuous flow for 300 min. Then, rCatcher-GFP (left column) and rGFP (right column) were added and the flow was maintained during 2 h with the recombinant proteins. Next, channels were washed with PBS during 90 min to remove the background fluorescence. **b** Images of GFP fluorescence and the differential interference contrast (DIC) at different times are shown. *S. aureus* Bap-Spy was grown in microfluidic plates and were incubated with rCatcher-GFP (left column) and rGFP (right column). Images were recorded every 30 min in separate fields to avoid photobleaching. Scale bar of panels represents 5 μm.

We next wondered whether the SpyCatcher can be also immobilized on the surface of a SpyTag-expressing bacteria growing under continuous flow. Using time-lapse microscopy, we monitored the recognition process that occurs between the two components of the system at single cell level. For that, *S. aureus* expressing Bap-Spy protein was grown in CellAsic microfluidic plates at 37 °C with a continuous flow of LB for 5 h (Fig. 6a). Thereafter, media was supplemented with rCatcher-GFP or rGFP (as negative control) and the flow resumed during 2 h. Finally, to remove background fluorescence from the system, the microfluidic channels were washed with PBS. The Fig. 6b shows that rCatcher-GFP binds SpyTag expressed at the bacteria cell surface even under flow conditions creating the already observed “cellular honeycomb”. When rGFP was added as negative control, no fluorescence was observed since there was no SpyCatcher to bind the SpyTag counterpart (Fig. 6b). Altogether these results point out the potential utility of Bap-Spy protein as a powerful biotechnological tool for the immobilization of epitopes using SpyCatcher at the cell surface of the bacteria or in the biofilm matrix of *S. aureus* grown under both static and flow conditions.

### Functionalization of *S. aureus*, *E. faecalis* and *L. monocytogenes* using recombinant engineered amyloid domains

**Fig. 7:**
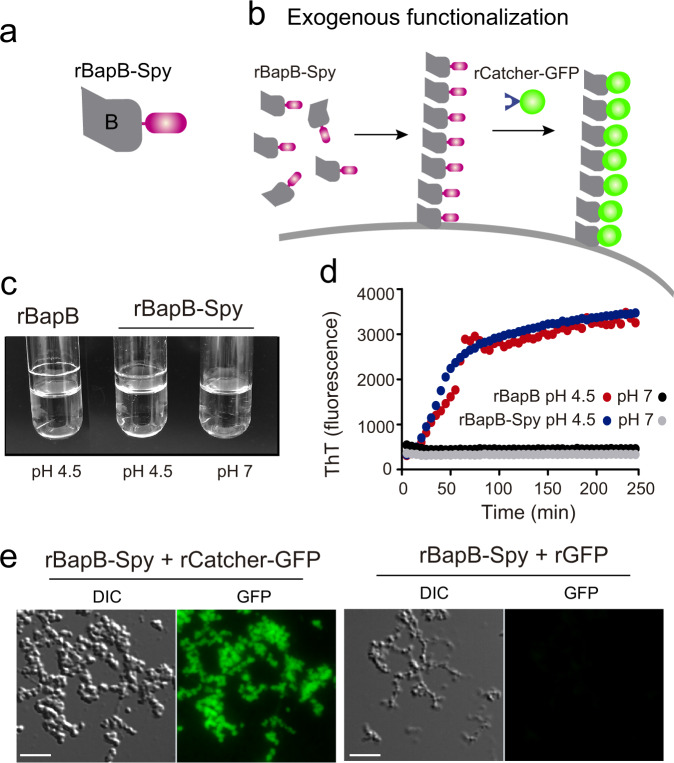
Recombinant rBapB-Spy protein forms aggregates under acidic conditions that can be functionalized with rCatcher-GFP. Schematic illustration showing **a** rBapB-Spy and **b** functionalized fibers after exogenous complementation with rBapB-Spy. **c** rBapB-Spy aggregates under acidic conditions in a similar way of rBapB. 2 μM of purified recombinant proteins (rBapB-Spy and rBapB) were incubated in phosphate-citrate buffer at pH 4.5 and pH 7. Aggregates were only visible at acid pH. **d** rBapB-Spy and rBapB aggregation kinetics were monitored by following the changes in relative ThT fluorescence emission intensity. **e** Immunofluorescence showing rBapB-Spy aggregates probed with purified recombinant rCatcher-GFP and purified rGFP alone (used as a control). The fluorescence of GFP and the differential interference contrast (DIC) images are shown. Scale bar of panels represents 5 μm.

In all the previous examples, functionalization of the biofilm required the tag addition into the *bap* gene by a genetic manipulation of the target bacteria. We previously described that exogenous addition of the N-terminal domain of Bap (region B) induces bacterial clumping under acidic culture conditions^26^. We reasoned that a recombinant B region engineered with SpyTag (rBapB-Spy) (Fig. 7a) will anchor to the bacterial surface of biofilm negative bacteria when added exogenously. Therefore, functionalization of an extracellular matrix from any bacteria can be achieved using SpyCatcher partner without genetic manipulation.

First, we tested whether the inclusion of the SpyTag tag to the recombinant BapB fragment (rBapB-Spy) could affect its functionality. We tested the aggregation phenotype under acidic conditions (pH 4.5) compared to neutral conditions (pH 7). As expected, results showed that rBapB-Spy formed macroscopic aggregates attached to the glass tube under acidic conditions but not at neutral pH, similar to the native rBapB protein (Fig. 7c). We also showed that aggregation kinetics of rBapB-Spy was similar to the one observed for rBapB by monitoring the changes in relative Th-T fluorescence (Fig. 7d). Next, we assessed functionalization of rBapB-Spy aggregates using rCatcher-GFP partner tag. Aggregates formed in the presence of rCatcher-GFP were fluorescent in comparison with the aggregates incubated with rGFP alone where no SpyCatcher is present (Fig. 7e).

**Fig. 8:**
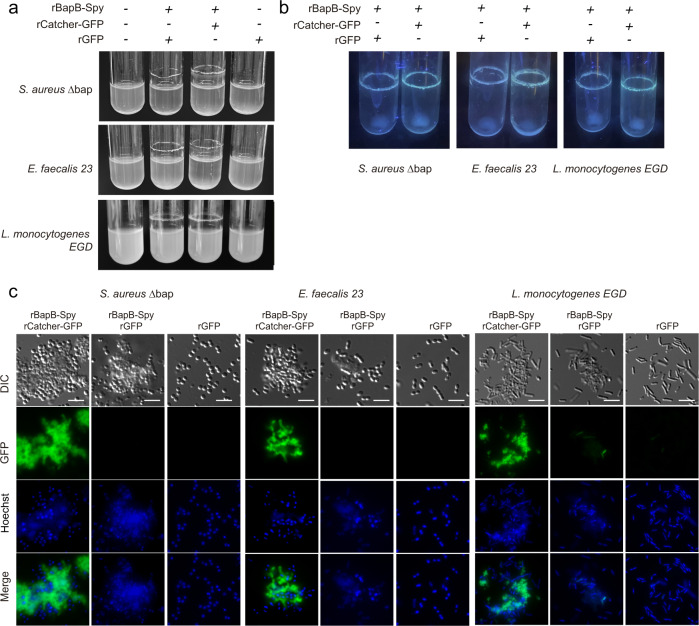
Functionalization of bacteria through exogenous complementation with rBapB-Spy. **a** Exogenous complementation of biofilm negative bacteria *S. aureus* Δ*bap, E. faecalis 23 and L. monocytogenes EGD* strains with rBapB-Spy coupled to purified recombinant rCatcher-GFP and purified rGFP alone (used as a control). **b** Fluorescence under uv-light of the rings formed by *S. aureus* Δ*bap, E. faecalis 23* and *L. monocytogenes EGD* cells when they are incubated with rBapB-Spy coupled to purified recombinant rCatcher-GFP and purified rGFP. **c** The fluorescence of GFP and Hoechst, the combination of both signals (merge panels) and the differential interference contrast (DIC) images are shown. Scale bar of panels represents 5 μm.

Having confirmed the rBapB-Spy functionality, we use this strategy to functionalize heterologously Gram-positive bacteria unable to form neither amyloid fibers nor biofilms. For that, we choose a *S. aureus* ∆*bap* and two bacteria negative for Bap-related genes such as *E. faecalis* 23 and *L. monocytogenes EGD* strain^26,29^. We exogenously complemented the *S. aureus* ∆*bap*, *E. faecalis 23 and L. monocytogenes EGD* strains with rBapB-Spy for their functionalization with rCatcher-GFP. As shown in Fig. 8a, the culture of *S. aureus* ∆*bap*, *E. faecalis 23 and L. monocytogenes EGD* strains in the presence of rBapB-Spy in a glucose-supplemented medium induced bacteria aggregation. Microscopic visualization of the rings formed on the glass tubes showed the formation of aggregates with bacteria embedded in them (Fig. 8b, DIC panel). These aggregates were decorated with fluorescence using rCatcher-GFP while no emission was detected in aggregates incubated with rGFP (Fig. 8b). These findings demonstrated that the exogenous addition of an engineered Bap protein can be used to promote bacterial aggregation in a functionalized biofilm matrix. This opens the possibility to create functionalized biofilm matrices without genetic modification of the target microorganisms.

## Discussion

Recently, novel strategies have been developed to create biofilms as artificial platforms for self-assembling functional tags^32,34,35^. One of these approaches is based on engineering extracellular matrix components, focusing on amyloids as potential targets^8,9,11^. In biofilms, bacteria secrete a wide variety of molecules that assemble to form the extracellular matrix, including polysaccharides, proteins, and nucleic acids^36^. Engineering the functionality of the extracellular polysaccharides requires complex pathway modifications to obtain chemically modified sugars. In contrast, the self-assembly capacity of amyloidogenic proteins, together with the stability and robustness of the nanofibers they generate, converts amyloids into protein complexes with exceptional properties for their manipulation and construction of functional biofilms with applications in different fields of nanotechnology, biotechnology and biomedicine^32^. In this regard, the most widely used amyloid protein has been CsgA from *E. coli.* Functional peptides are fused to the monomeric building block, CsgA, which is exported to the extracellular space by a dedicated machinery and self-assembly into engineered fibers^16–20^. Resulting fibers exhibit properties including specific surface adhesion, molecular recognition, electron transfer or catalysis^11,12^. In the same way, the amyloid protein TasA has also been engineered for conferring living glues properties to *B. subtilis* biofilms^35^ and printing of this functionalized biofilm was performed in 3D^9^. Moreover, other groups have focused on the use of amyloids in bioremediation^16^, biosensing^37^ or clearance of pathological amyloids found in nature^38^, among other applications.

Here, we explore the dual function of the facultative amyloid Bap to confer to the bacterial cell surface or the amyloid fibers comprising the biofilm additional purposes. Bap is a multidomain protein with a Sec-dependent signal peptide for externalization, a N-terminal domain which comprises the amyloidogenic B region and a core domain of repeats^24,31^. This protein is a simple model of amyloid formation, which does not require an accompanying system for its expression and secretion and allows coupling proteins and peptide of various lengths and secondary structures without affecting Bap functionality (Fig. 1). Indeed, Bap amyloidogenic potential is concentrated in its region B, which is processed and self-assemble autonomously into amyloid fibers without the requirement of any accessory proteins^26^. In contrast, although CsgA is able to polymerize on its own upon prolonged incubation, it requires accessory gene products to accelerate CsgA polymerization into amyloid fibers^39,40^. Analogously, TasA protein is secreted as a monomer and requires not only pH acidification, but also a conformational change to assemble into amyloid fibers, normally assessed by TapA and SipW accessory proteins that help in the polymerization process^41,42^. Since short peptides rather than full length proteins are suitable as building blocks^37^, Bap represents an ideal model for engineering biofilm matrix components. Region B of Bap is self-sufficient to induce aggregation and biofilm phenotype. It contains two short amyloidogenic peptides, peptide I _487_TVGNIISNAG_496_ and peptide II _579_GIFSYS_584_^26^. At acidic pH (pH < 5), region B of Bap suffers a conformational change and acquires an amyloidogenic state that results in the formation of fibrillar structures that mediate biofilm formation^26^. Therefore, the amyloid architecture of region B facilitates a self-assembled nanofiber mesh, while the fused peptide/protein domains of this module permit the properties of the resulting biofilm to be altered in a programmable way. In addition, by modulating pH, it is possible to control the assembly or disassembly of the Bap-mediated biofilm. We have shown that functionality of tagged Bap proteins was influenced by pH fluctuations, as observed for the native Bap. Moreover, pH could be used to voluntarily control the formation of programmed biofilms as we demonstrated using cells expressing Bap-mCherry (Figs. 3 and 4).

The proof-of-concept of SNAP-tag/SNAP-surface system, in which a recognition process takes part, opens the possibility to use other versatile systems. We have functionalized Bap with GFP protein using the SpyTag/SpyCatcher system (Fig. 5). We chose this technology because they form a robust covalent bond with the ability to form site-specific attachment between the two modules, even in a complex mixture^20^. In fact, the versatility of this system could be advantageous in enzyme immobilization for biocatalysis, electron transport or the display of arbitrary proteins with variable length^11,43^. Our results confirm that the SpyTag can be fused to Bap maintaining the capacity to immobilize its partner tag SpyCatcher after extracellular assembly of Bap into amyloid fibers (Fig. 6). Although we coupled GFP to SpyCatcher as a proof of concept for Bap functionalization process, we anticipate that different Catcher-tagged constructs could be used for biofilm functionalization such as nanobodies able to recognize epitopes of interest^44^, catalytic enzymes with industrial applications^20^, quantum dots, gold nanoparticles^10^ or even virus-like particles for the development of synthetic vaccines^45^.

Another important aspect of this work is that we have functionalized a biofilm without any genetically modification of the bacteria (Fig. 8). In some works employing TasA with engineered fusion peptides, the functionalization was reached by externalizing the protein from the chromosome or from plasmid-complemented expression, but no external recombinant TasA was used^9,35^. Here we produced recombinantly the amyloidogenic region of Bap with the SpyTag peptide. Exogenous complementation of Gram-positive bacteria with an engineered B-region of Bap promoted a multicellular behaviour phenotype (Fig. 8). The use of non-genetically modified functionalized bacteria can be considered as the main advantage of this technology for its use as a therapeutic tool.

In short, taking advantage of Bap externalization as an adhesin and polymerization into amyloid-like fibers, we can secrete a wide variety of Bap-peptide constructs that will be part of the extracellular matrix. The use of facultative amyloids as an immobilization tool in biofilms has several benefits. Bacteria can be engineered to produce functional domains and manipulate the transition from the adhesin conformation to amyloid conformation by controlling parameters such as temperature and pH. Under unfavorable environmental conditions, the amyloid matrix preserves the stability of functional domains for longer periods of time. The assembly of functionalized subunits into amyloid fibers allows the accumulation of functional domains at the nanometric scale. A warning for the use of amyloids to functionalize biofilm matrices is that cross-seeding between the bacterial amyloids and human aggregation-prone proteins can occur^46^. Human and bacterial amyloids vary widely with respect to their amino acid sequences but all share a similar tertiary structure^47^. Thus, it cannot be excluded that bacterial amyloids could serve as nucleators of human amyloids and trigger undesirable aggregation responses in the host^48^. This can limit the biomedical applications of functionalized biofilms. However, the controlled switch between two functional conformations of the same protein domain could be useful in other non-health related biotechnological applications.

## Methods

### Bacterial strains and growth conditions

Strains and plasmids are listed in Supplementary Table 1. Oligonucleotides used in the study are shown in Supplementary Table 2 and were synthesized by StabVida (Caparica-Portugal). Enzymes for DNA manipulation were used according to manufacturer’s recommendations (Thermo Scientific). *S. aureus* were grown in Luria-Bertani (LB) broth. To archive acidic conditions at stationarity phase *S. aureus* were grown in LB supplemented with 0.5% w/v glucose (LB-glu). The colony morphology of *S. aureus* V329-labelled cells was analyzed using Congo red agar plates (30 g/L TSB, 15 g/L agar, 0.08% (w/v) CR, 20 g/L sucrose). Plasmids were transformed into staphylococci by electroporation. Briefly, *S. aureus* cells were cultured in B2 broth (casein hydrolysate 10 g/l, yeast extract 25 g/l, NaCl 25 g/l, K_2_HPO_4_ 1 g/l, glucose 5 g/l) until the OD at 650 nm reaches 0.5. Cells were washed three times with glycerol 10% (v/v). Cells were resuspended in 15 ml of glycerol 10% (v/v) and incubated at 20 °C for 15 min. After incubation, cells were resuspended in 10% glycerol at the final concentration of 1 × 10^10^ CFU/ml. For staphylococcal electroporation, 50 μl of the electrocompetent cells were mixed with 5 μg of plasmid and the mix was added to an ice-cold 0.1-cm gap electrode cuvette. One pulse was given at 100 Ω, 1.25 V, and 25 F capacity. Cells were plated onto TSA (trypticase soy agar) with erythromycin (Em), 1.5 μg/ml.

### Chromosomal labelling of Bap with functional peptides/proteins

6xHis-tag (6 amino acids, aa), SpyTag (13 aa), Mefp3-tag (48 aa), MT1-tag (61 aa), SNAP-tag (181 aa) and mCherry-tag (235 aa) were fused to domain B of Bap (between amino acids 819 and 820). For 6xHis-tag two regions of Bap were amplified using Phusion® High-Fidelity DNA Polymerase (Thermo Scientific): (i) the first comprised the last 477 bp of the region B of Bap and was amplified using primers Bap_SNAP_A_EcoRI and BapB_B_HIS_rev2, in which the latter is included the 6xHis amino acid sequence, (Supplementary Table 2); (ii) second fragment comprised the first 414 bp of the C core repeat region of Bap that was amplified using primers BapB_C_HIS_fw and Bap_SNAP_D_BamHI. Overlapping PCR was performed using primers Bap_SNAP_A_EcoRI and Bap_SNAP_D_BamHI. For SpyTag two regions of Bap were amplified: (i) first fragment comprised the 477 bp of the region B of Bap (primers Bap_SNAP_A_EcoRI and Bap_Spy_B, Supplementary Table 2); (ii) second fragment comprised the ∼414 bp of the C core repeat region (primers Bap_Spy_C and Bap_SNAP_D_BamHI). SpyTag sequence (39 bp) was obtained by the annealing of primers Spy_tag_fw and Spy_tag_rv (Supplementary Table 2) after denaturing them at 96 °C for 15 min. Overlapping PCR was performed using Bap_SNAP_A_EcoRI and Bap_SNAP_D_BamHI. For SNAP-tag we amplified a fragment comprising the last 477 bp of region B of Bap using primers Bap_SNAP_A_EcoRI and Bap_SNAP_B, a fragment comprising the first ∼414 bp of the C core region using primers Bap_SNAP_C and Bap_SNAP_D_BamH and the *snap-tag* sequence that was PCR amplified from plasmid pSNAP-tag® (T7)-2 (Supplementary Table 1) using primers SNAPtag_A and SNAPtag_B. Overlapping PCR was performed to obtain a single fragment using primers Bap_SNAP_A_EcoRI and Bap_SNAP_D_BamHI. For Mefp3-tag we amplified a first fragment corresponding to the last ∼477 bp of the B region (primers Bap_SNAP_A_EcoRI and Bap-Mefp3_B), a fragment corresponding to the first ∼414 bp of the C core region (primers Bap-Mefp3_C and Bap_SNAP_D_BamHI). The *mefp3* sequence was synthetized by GeneArt Gene Synthesis (Invitrogen) and was PCR amplified using primers Mefp3-Fw and Mefp3-Rv. Overlapping PCR was performed to obtain a single fragment using primers Bap_SNAP_A_EcoRI and Bap_SNAP_D_BamHI. For MT1-tag we amplified: a fragment comprising the last ∼477 bp of the B region using primers Bap_SNAP_A_EcoRI and Bap_Oro_B, a fragment comprising the first ∼414 bp of the C core region using primers Bap_Oro_C and Bap_SNAP_D_BamHI and metallothionein-I was amplified from pMAL-c2x-MT1 plasmid (Supplementary Table 1) using primers Oro_A and Oro_B. Overlapping PCR was performed using primers Bap_SNAP_A_EcoRI and Bap_SNAP_D_BamHI. For mCherry-tag we amplified: a fragment corresponding to the last ∼477 bp of the B region (primers Bap_SNAP_A_EcoRI and Bap-mCherry-B); a fragment corresponding to the first ∼414 bp of the C core region (primers Bap-mCherry-C and Bap_SNAP_D_BamHI). The *mCherry* sequence was PCR amplified using primers mCherry-pHRR-Fw and mCherry-pHRR-Rv from plasmid pHRR (Supplementary Table 1). Overlapping PCR was performed t using primers Bap_SNAP_A_EcoRI and Bap_SNAP_D_BamHI. All fused fragments were purified and cloned using *Eco*RI and *Bam*HI enzymes into the shuttle vector pMAD (Supplementary Table 1). The resulting plasmids were transformed into *S. aureus* V329 by electroporation and tags were incorporated onto Bap sequence by homologous recombination.

### Multicellular behaviour phenotypes

To evaluate Bap functionality with the different peptide/protein labels, we assessed the multicellular behavior of *S. aureus* V329 in both cell-to-cell aggregation and biofilm-forming conditions. In the former case, overnight cultures were grown in LB and LB-glu at 37 °C and 200 rpm and macroscopically observed for the presence or absence of aggregates adhered to the glass tube or at the bottom of the tube (clumping). In the latter case, we performed the biofilm formation assay by diluting 1:40 the overnight cultures (OD_600nm_ = 5) in 200 μl of LB or LB-glu in a 96-well polystyrene microtiter plate. Plates were incubated for 18 h under static conditions at 37 °C. Wells were washed and biofilms formed on the microtiter wells were stained with crystal violet (0.1% v/v). The colorant was solubilized with alcohol-acetone (80 v:20 v) and the OD595nm was determined using a microplate reader (Synergy H1 Hybrid Multi-Mode Reader). Each condition was performed in triplicate. Statistical significance differences were determined using non-parametric one-tail Mann Whitney test: *P < 0.05. Colony morphology of *S. aureus* V329-labeled strains was visualized on Congo Red (CR) agar plates. Overnight cultures were grown in LB-glu. Ten μl of each culture was plated in duplicate and phenotype was observed in a stereo microscope (Zeiss).

### Construction of recombinant proteins

To create the expression plasmid for rBapB-Spy, region B of Bap was amplified from *S. aureus* Bap-Spy using Bap-LIC-Fw and Bap-LIC-Rv primers (Supplementary Table 2) designed for use in the pET46-Ek/LIC vector (Novagen). To plasmid for rGFP expression, the *gfp* sequence was PCR amplified from pAD-GFP plasmid (Supplementary Table 1) using primers GFP_Ek_LIC_Fw and GFP_Ek_LIC_Rv (Supplementary Table 2). The resulting 717 bp fragment was cloned into the pET46-Ek/LIC vector (Novagen). To obtain the plasmid for rCatcher-GFP expression plasmid, the *gfp* sequence amplified from pAD-GFP plasmid and including the NdeI-SalI restriction sites was fused to the *spycatcher* sequence (SalI /EagI) and it was cloned into the pUA1108 plasmid (Supplementary Table 1). *Gfp*-*spycatcher* sequence was PCR amplified from pAU1108-GFP-Catcher plasmid (Supplementary Table 1) using primers GFP_Ek_LIC_Fw and GFP_Catcher_Ek_LIC_Rv (Supplementary Table 2). The resulting 1095 bp fragment was cloned in pET46-Ek/LIC vector (Novagen). Constructs resulted expression plasmids of recombinant proteins rGFP, rCatcher-GFP and rBapB-Spy.

### Expression and purification of recombinant proteins

Overnight cultures of *E. coli* BL21 Origami (DE3) containing pET46-Ek/LIC plasmids (Supplementary Table 1) were diluted 1:100 and grown to an OD_600nm_ of 0.5 in LB supplemented with 100 μg/ml ampicillin and 1% (w/v) glucose, at 37 °C and 200 rpm. Before induction, glucose from the media was removed and replaced by LB with 100 μg/ml ampicillin. Isopropyl β-D-thiogalactopyranoside (IPTG) was added to a final concentration of: 0.1 mM for rBapB-Spy, 1 mM for rGFP-SpyCatcher and 1 mM for rGFP. The cultures were shaken (150 rpm) at 20 °C overnight. Pellets were resuspended in BugBuster lysis buffer (Novagen) and incubated 30 min RT. Cells were sonicated (3 cycles of 30 s pot 4; 5 cycles of 30 s pot 5) and centrifuged (26,200 × *g*, 30 min and 4 °C). Supernatants were filtered (0.45 μm). Recombinant proteins were purified by Ni affinity chromatography using HisGraviTrap gravity-flow columns (GE Healthcare) following the manufacturer’s protocol. Detection of recombinant proteins cloned in pET46-Ek/LIC vector was assessed using mouse monoclonal HRP-conjugated anti-polyHistidine antibody (1:2000, Sigma). The concentration of purified proteins was determined using the Bradford Protein Assay (Bio-Rad) with BSA protein as a standard.

### Labelling of Bap-SNAP fusion protein

Overnight culture of *S. aureus* V329-SNAP was diluted 1:100 and grown in LB and LB-glu. Samples were obtained at OD_600nm_ of 0.5, 1, 3, and 5. Cells were harvested and washed 3 times with phosphate-buffered saline (PBS). Bacteria were incubated with SNAP-surface^®^ Alexa fluor^®^ 488 substrate (New England BioLabs, Inc) at 37 °C, 200 rpm for 30 min. To remove the dye, cells were washed three times with PBS and fixed with paraformaldehyde (3% v/v) for 10 minutes at room temperature. After several washing with PBS, cells were stained with 1:1000 diluted Hoechst (Thermo Scientific).

### Labelling of Bap-Spy fusion protein

For the labelling of Bap-Spy fusion protein, overnight culture of *S. aureus* Bap-Spy was diluted 1:100 and grown in LB and LB-glu. Samples were obtained at OD_600nm_ of 0.5 and 1. Cells were harvested and washed twice with PBS-0.5% (v/v) Tween. Bacteria were resuspended in PBS-Tween 0.5% (v/v) and BSA 1% (w/v) at final OD_600nm_ = 1. Cells were incubated with purified rGFP and rCatcher-GFP (0.5 mg/ml) at 37 °C, 200 rpm and light-protected for 1 hour. After three washes with PBS-Tween 0.5% (v/v), the samples were fixed with 3% (v/v) paraformaldehyde for 10 min at room temperature. After several washes with PBS, cells were stained with 1:1000 diluted Hoechst (Thermo Scientific) to label nucleoids and mounted onto glass slides.

### *S. aureus* V329-mCherry fusion protein assay

Overnight culture of *S. aureus* V329-mCherry was diluted 1:100 and grown in LB and LB-glu. Samples were obtained at OD_600nm_ of 0.5 and 5. Cells were fixed with 3% (v/v) paraformaldehyde for 10 min at room temperature. After washing the cells with PBS, they were stained with 1:1000 diluted Hoechst (Thermo Scientific) and mounted onto glass slides.

### Fluorescence microscopy procedures

Images from preparations were acquired using a Leica DMi8 fluorescence microscope equipped with a Leica HCX PL APO 100×/1.40–0.70 Oil objective, a Hamamatsu ORCA Flash 4.0 LT camera and the LAS X software. Images from the green, blue, red and differential interference contrast (DIC) channels were processed using the Icy software (http://icy.bioimageanalysis.org/) and Adobe Photoshop software packages.

### Time-lapse fluorescence microscopy

Single cell assay for monitoring mCherry and GFP fluorescence was carried out by time-lapse fluorescence microscopy using microfluidic plates. Overnight cultures in LB of *S. aureus* V329 Bap-mCherry and Bap-Spy were diluted to an initial OD_600nm_ of 0.03 in LB and grown at 37 °C and 200 rpm until OD_600nm_ of 0.5. Bacteria were diluted 1:10 and loaded into B04A microfluidic plates (ONIX, CellASIC) following the manufacturer’s instructions. Microfluidic plates were placed into the OKO-lab chamber of a fully-automatized Leica DMi8 fluorescence microscope and incubated at 37 °C. In the assay with *S. aureus* Bap-mCherry, the initial media (LB) was maintained with a flow rate of 2 psi during 5.5 h and then switched to acidified LB, previously obtained from stationary LB-glu cultures in the second channel (the first channel remains with LB) with 2 psi flow rate until the end of the image acquisition.

In the case of SpyTag/SpyCatcher assay, the initial media (LB) was maintained with a flow rate of 2 psi during 5 h. After that, rCatcher-GFP and rGFP were added during 1.5 h until a final wash of PBS for 90 min. Media switching and flow rate settings were controlled using the CellASIC ONIX FG Software (v 5.5.1.0). Images from four different fields of each microfluidic chamber were acquired every 30 mins using a Leica HCX PL APO 100×/1.40–0.70 Oil objective and the LAS X software during 8 h. Images from the fluorescent proteins and differential interference contrast (DIC) channels were processed using the Icy bioimage software for bioimage analysis (http://icy.bioimageanalysis.org).

### Quantification of the fluorescence intensity

Quantification of the fluorescence signal at the single cell level was performed using the Intensity Profile plugin of the Icy-software. This plugin calculates the intensity value of each pixel along a cross-section line of 3 μm that was drawn centered on individual cells. The mean of the intensity values (*n* = 20 to 40) was plotted to compare the distribution of the fluorescence intensity along the region of interest.

### Formation of rBapB-Spy aggregates

Recombinant rBapB and rBapB-Spy proteins (2 μM) were incubated in phosphate-citrate buffer at acidic pH (pH 4.5) and neutral pH (pH 7). Protein aggregates were visualized as a macroscopic ring adhered to the glass wall after incubation under shaking conditions (200 rpm) for 24 h at 37 °C. To functionalize rBapB-Spy with rCatcher-GFP, 2 μM of rBapB-Spy were incubated with rCatcher-GFP (0.5 mg/ml) or rGFP (0.5 mg/ml) in phosphate-citrate buffer at acidic pH (pH 4.5) at 37 °C and 200 rpm for 24 h. Protein aggregates that formed a visible ring on the glass tubes were collected by centrifugation, washed twice with PBS and mounted onto glass slides.

### Aggregation kinetics

Aggregation kinetics of recombinant rBapB and rBapB-Spy proteins (0.4 mg/ml) in phosphate–citrate buffer at pH 4.5 and pH 7 in the presence of 50 μM Th-T were recorded for 250 min under agitation (800 rpm) at 25 °C. The kinetic points were measured exciting at 440 nm and emission was recorded at 475 nm; 5 nm slit widths were used for excitation and emission in a Synergy H1 Hybrid Multi-Mode Reader.

### Exogenous functionalization

Functionalization of genetically non-modified bacteria was performed using *S. aureus* ∆*bap*, the *esp* negative strain *E. faecalis* 23 and *Listeria monocytogenes* EGD. *S. aureus* ∆*bap* and *E. faecalis* 23 were grown overnight in LB-glu with rBapB-Spy (2 μM), rBapB-Spy (2 μM) supplemented with rCatcher-GFP (0.5 mg/ml) and rBapB-Spy (2 μM) incubated with rGFP (0.5 mg/ml). The bacterial clumps formed in each tube were collected by centrifugation, washed twice with PBS and samples were mounted onto glass slides. Preparations were observed, acquired and processed the same way as before.

## Supplementary information

Supplementary material

## Data Availability

The data that support the findings of this study are included in the article, its supplementary information files, or are available from the corresponding author upon reasonable request.
