# Supplementary material for: Bacterial biofilm functionalization through Bap amyloid engineering
Source: NPJ Biofilms Microbiomes. 2022 Aug 1;8:62. doi: 10.1038/s41522-022-00324-w (PMC9339546; doi:10.1038/s41522-022-00324-w)
Supplement: Supplementary file 1 — Supplementary material [file 41522_2022_324_MOESM1_ESM.pdf]

Supplementary information

Supplementary Table 1. Bacteria and plasmids

**Strain name**	**Relevant characteristics**	**Source of reference**
V329	Expresses Bap. Biofilm positive.	[1]
V329_His	6xHis tag cloned at the end of B region of Bap	This study
V329_SpyTag	SpyTag cloned at the end of B region of Bap	This study
V329_Mefp3	Mefp3 gene cloned at the end of B region of Bap	This study
V329_MT1	MT1 gene cloned at the end of B region of Bap	This study
V329_SnapTag	SnapTag gene cloned at the end of B region of Bap	This study
V329_mCherry	mCherry gene cloned at the end of B region of Bap	This study
BL21 (DE3)	F^- *ompT* *hsd*S_B(r_B^-, m_B^-) *gal dcm lon λ(DE3 [lacI lac*UV5-T7 *gene1 ind1 sam7 nin5])*	Novagen
BL21(DE3)+pET46-ek/LIC:*bap_B:*SpyTag	BL21 carrying pET46 for overexpression of Bap_B protein fused to SpyTag protein	This study
BL21(DE3)+pET46-ek/LIC: *gfp:*SpyCatcher	BL21 carrying pET46 for overexpression of GFP protein fused to SpyCatcher protein	This study
BL21(DE3)+pET46-ek/LIC*:*SpyCatcher	BL21 carrying pET46 for overexpression of SpyCatcher protein	This study
BL21(DE3)+pET46-ek/LIC:*gfp*	BL21 carrying pET46 for overexpression of GFP protein	This study
E. faecalis 23	esp-, non-biofilm forming	[2]
V329 Δ*bap*	V329 with deletion in *bap* gene	[3]
**Plasmid name**	**Description**	**Source of reference**
pJET1.2/blunt	Cloning vector	Termo Scientific
pET46-ek/LIC	Expression vector	Novagen
pSNAP-tag®(T7)-2	Expression vector encoding the SNAP-tag protein	New England Biolabs
pUA1108-GFPCatcher	Expression vector encoding *gfp-spycatcher* sequence under the expression of P*tac* promoter	This study
pMAL-c2x-MT1	Plasmid used to amplify the metallothionein (MT1)	Adegene
pHRR	Plasmid used to amplify the *mcherry*	[4]
pET46-ek/LIC:*bap_B:*SpyTag	pET46 for induction of Bap_B protein fused to SpyTag protein	This study
pET46-ek/LIC: gfp:SpyCatcher	pET46 for induction of GFP protein fused to SpyCatcher protein	This study
pET46-ek/LIC: SpyCatcher	pET46 for induction of SpyCatcher protein	This study
pET46-ek/LIC:*gfp*	pET46 for induction of GFP protein	This study
pMAD	*E. coli - S. aureus* shuttle vector with a thermosensitive origin of replication for Gram-positive bacteria. The vector contains the *bga*B	[5]
pMAD:6xHis	pMAD for integrating 6xHis tag in the chromosome	This study
pMAD:SpyTag	pMAD for integrating SpyTag tag in the chromosome	This study
pMAD:Mefp3	pMAD for integrating Mefp3 tag in the chromosome	This study
pMAD:MT1	pMAD for integrating MT1 tag in the chromosome	This study
pMAD:SnapTag	pMAD for integrating SnapTag tag in the chromosome	This study
pMAD:mCherry	pMAD for integrating mCherry tag in the chromosome	This study

Supplementary Table 2. Oligonucleotides and plasmids used in this study

Construction	Primer name	Sequence 5’-3’
Bap-Snap	Bap_SNAP_A_EcoRI	5’-GAATTCGAAACTGTAGGCGTTAGAGT
	Bap_SNAP_B	5’-GGTGGTGCGCTTCATTTCGCAGTCTTTGTCCATATTTACAGTTGCTGTACCAAC
	Bap_SNAP_C	5’-GAGGGCCACAGACTGGGCAAGCCTGGGCTGGGTTTCAGATGGTACATTCTCAGTG
	Bap_SNAP_D_BamHI	5’-GGATCCTGTATTTACTTCATCTAATGTTGG
	SNAPtag_A	5’-TTGGTACAGCAACTGTAAATATGGACAAAGATTGCGAAATGA
	SNAPtag_A	5’ACACTGAGAATGTACCATCTGATCCCAGACCCGGTTTACCCAG
Bap-HIS	BapB_B_HIS_rev2	5’-GTGGTGATGGTGATGATGATTTACAGTTGCTGTACCAACTGTTGTACCCT
	BapB_C_HIS_fw	5’-CATCATCACCATCACCACTCAGATGGTACATTCTCAG
Bap-Spy	Bap_Spy_B	5’-CTTCGTCGGCTTGTAGGCGTCCACCATCACGATGTGGGCATTTACAGTTGCTGTACCAAC
	Bap_Spy_C	5’-GCCCACATCGTGATGGTGGACGCCTACAAGCCGACGAAGTCAGATGGTACATTCTCAGT
	Spy_tag_fw	5’-GGGCCAGCCGGTACCGCCCACATCGTGATGGTGGACGCCTACAAGCCGACGAAG
	Spy_tag_rv	5’-GGGCCAGCCGGTACCCTTCGTCGGCTTGTAGGCGTCCACCATCACGATGTGGGC
Bap-Mef3	Bap-Mefp3-B	5’-CGGGCCATAGTAATCCGCATTTACAGTTGCTGTACCAAC
	Bap-Mefp3-C	5’-AGACGCGGCAAATACTGGTTCAGATGGTACATTCTCAGTG
	Mefp3-Fw	5’-GCGGATTACTATGGCCC
	Mefp3-Rv	5’-CCAGTATTTGCCGCGT
Bap-mCherry	Bap-mCherry-B	5’-TCTTCTTCACCTTTACTAGTATTTACAGTTGCTGTACCAAC
	Bap-mCherry-C	5’-GGTATGGATGAATTATACAAAGATGGTACATTCTCAGTGTCA
	mCherry-pHRR-Fw	5’-ACTAGTAAAGGTGAAGAAGATA
	mCherry-pHRR-Rv	5’-TTTGTATAATTCATCCATACCAC
Bap-MT1	Bap_Oro_B	5’-GGAGCAGTTGGGGTCCATATTTACAGTTGCTGTACCAAC
	Bap_Oro_C	5’-GTGCACGTGCTGTGCCTCAGATGGTACATTCTCAGT
	Oro_A	5’-TACAGCAACTGTAAATATGGACCCCAACTGCTCC
	Oro_B	5’-ACACTGAGAATGTACCATCTGAGGCACAGCACGTGCAC
rBapB_Spy	Bap-LIC-Fw	5’-GACGACGACAAGATGCAAAAATCTTTAGGTTACACAGATAATTATAC
	Bap-LIC-Rv	5’GAGGAGAAGCCCGGTTCTTCGTCGGCTTGTAGGCGTCCACCATCACGATGTGGGCGCCAGAACCACCG TGCCATTTACAGTTGCTGTACCAACTGTTGTAC
Catcher-Gfp	GFP_Ek_LIC_Fw	5’-GACGACGACAAGATGAGTAAAGGAGAAGAACTTTTC
	Catcher_EK-LIC_Rv	5’-GAGGAGAAGCCCGGTTAAATATGAGCGTCACCTTTAG
Gfp	GFP_Ek_LIC_Fw	5’GACGACGACAAGATGAGTAAAGGAGAAGAACTTTTC
	GFP_Ek_LIC_Rv	5’GAGGAGAAGCCCGGTTATTTGTATAGTTCATCCATGCC

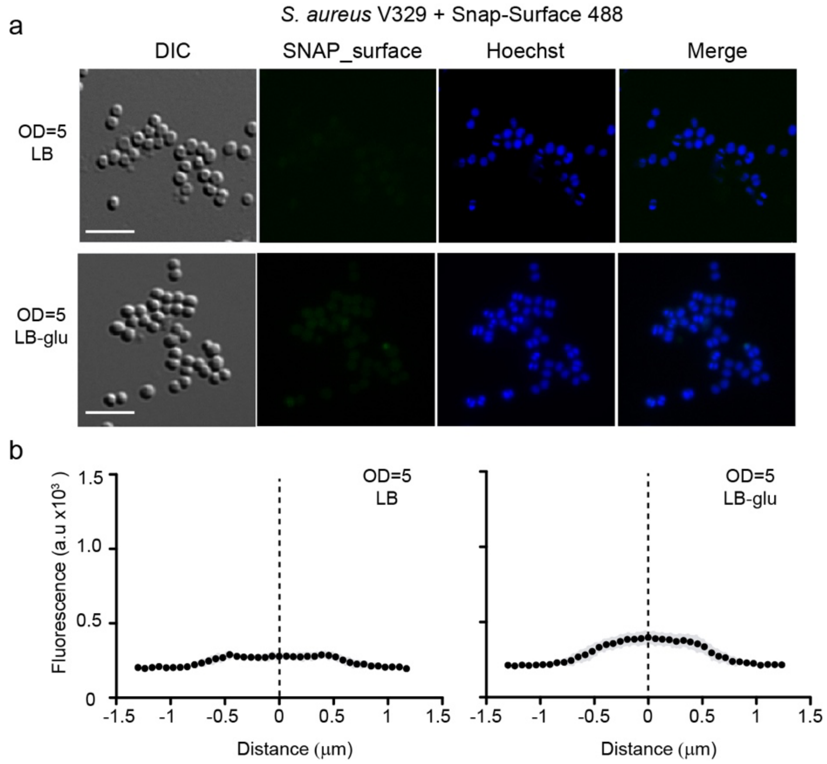

Supplementary Figure 1: Negative control for SNAP fluorescence. a) *S. aureus* expressing the Bap without SNAP-tag was grown in LB and LB-glu until stationary phase (OD600=5). Cells were labelled with SNAP-surface 488 substrate and Hoechst. The fluorescence of SNAP-surface 488 and Hoechst, the combination of both signals (merge panels) and the differential interference contrast (DIC) images are shown. Scale bar of panels represents 5 µm. b) The fluorescence intensity was determined using the Intensity profile plugin of the Icy-software. Graphs correspond to the mean of the intensity profiles of cross-sections cells (n=20). Gray shadow corresponds to the standard deviation of the mean.

Supplementary references.

1. Toledo-Arana, A. *et al.* The enterococcal surface protein, Esp, is involved in *Enterococcus faecalis* biofilm formation. *Appl Environ Microbiol* **67,** 4538–4545 (2001).
2. Valle, J. *et al.* Bap, a Biofilm Matrix Protein of Staphylococcus aureus Prevents Cellular Internalization through Binding to GP96 Host Receptor. *PLoS Pathogens* **8,** e1002843 (2012).
3. Medina-Ruiz, L. *et al.* Overexpression of the recA gene decreases oral but not intraperitoneal fitness of Salmonella enterica. *Infection and Immunity* **78,** 3217–3225 (2010).
4. Bastet, L. *et al.* Regulation of Heterogenous LexA Expression in Staphylococcus aureus by an Antisense RNA Originating from Transcriptional Read-Through upon Natural Mispairings in the sbrB Intrinsic Terminator. *Int J Mol Sci* **23,** 576 (2022).
5. Arnaud, M., Chastanet, A., & Débarbouillé, M. (2004). New vector for efficient allelic replacement in naturally nontransformable, low-GC-content, gram-positive bacteria. Applied and Environmental Microbiology, 70(11), 6887–6891.
